# COVID-19-Induced Diabetic Ketoacidosis in an Adult with Latent Autoimmune Diabetes

**DOI:** 10.7759/cureus.12690

**Published:** 2021-01-13

**Authors:** Yetunde B Omotosho, Grace W Ying, Mark Stolar, Arvin Junn P Mallari

**Affiliations:** 1 Internal Medicine, Chicago Medical School Internal Medicine Residency Program at Northwestern McHenry Hospital, McHenry, USA; 2 Endocrinology, Diabetes and Metabolism, Northwestern University Feinberg School of Medicine, Chicago, USA

**Keywords:** autoimmune diabetes, covid-19 and diabetes, coronavirus disease 2019 (covid-19), latent autoimmune diabetes in adults (lada)

## Abstract

Latent autoimmune diabetes in adults (LADA) is a type of slow-onset, immune-mediated insulin deficiency involving progressive destruction of beta-cell function. Despite sharing some similarities with both type 1 and type 2 diabetes, LADA is a separate entity that should be given equal attention as patients with this condition are subject to severe complications and preventable hospitalizations without proper medical management if not diagnosed in a timely manner. Herein, we describe the case of a 45-year-old Hispanic female with a past medical history of presumed noninsulin-dependent type 2 diabetes managed with metformin for six years who presented with fatigue, dry cough, and intermittent presyncope for one week. Laboratory data revealed evidence of diabetic ketoacidosis. She also tested positive for coronavirus disease 2019 (COVID-19) caused by the severe acute respiratory syndrome coronavirus 2 (SARS-CoV-2). Although her respiratory status was stable and did not require treatment for COVID-19, she required high doses of insulin to normalize hyperglycemia and spent two days in the intensive care unit (ICU). Further evaluation revealed positive islet autoantibodies and decreased C-peptide levels, leading to a diagnosis of LADA. SARS-CoV-2 has been shown to enter islet cells via the angiotensin-converting enzyme-2 (ACE2), causing damage and inducing acute diabetes and associated complications, including ketoacidosis. It is conceivable that this patient had acute worsening of her diabetes through this mechanism. Recognition of this association may contribute to the timely diagnosis of LADA and prevention of medical complications due to inappropriate diabetes therapy.

## Introduction

LADA is a disorder in which, despite the presence of islet antibodies at the time of diagnosis, the progression of autoimmune beta cell failure is slow. LADA patients are, therefore, not requiring insulin for at least the first six months after diagnosis [1]. However, close follow-up and prompt therapeutic management with insulin are essential once a complete loss of beta-cell function is reached, often manifesting as uncontrolled hyperglycemia. COVID-19 was declared a global pandemic on March 11, 2020 by the World Health Organization (WHO) [2]. The Cochrane Database of Systematic Reviews documents that COVID-19 has a wide range of symptoms, making it difficult to characterize the disease. Evolving data suggest that patients with COVID-19 and diabetes are more likely to associate with a severe or critical illness, leading to an increase in mortality likely due to low innate and humoral immunity in this patient population [3]. Severe acute respiratory syndrome coronavirus 2 (SARS-CoV-2) infection is mediated by the binding of its spike protein (S-protein) through a cellular receptor located on its target cells. A recent study proved that angiotensin-converting enzyme-2 (ACE2) is a functional receptor for the SARS-CoV-2 S-protein, thus facilitating viral entry into target cells [4]. Studies have confirmed ACE2 messenger ribonucleic acid (mRNA) expression, specifically in the bronchus, lung, ileum, testes, and some other cardiovascular, renal, gastrointestinal, and pancreatic tissues [1, 5]. The expression of ACE2 mRNA in the pancreas has been shown to induce islet cell damage and acute beta cell dysfunction presenting as new-onset diabetes in healthy individuals or worsening hyperglycemia or ketoacidosis in diabetic patients [5].

## Case presentation

A 45-year-old Hispanic female with a past medical history of non-insulin-dependent type 2 diabetes diagnosed six years earlier and gestational diabetes presented to our facility with a complaint of progressively worsening fatigue, nonproductive cough, and pre-syncope for one week. Associated symptoms included intermittent fever, chills, myalgia, and generalized weakness. Additionally, she denied having shortness of breath, chest pain, palpitations, hemoptysis, nausea, vomiting, recent illness, or sick contacts. Her only medication was metformin and with good overall adherence. She reported a good state of health without any complications from diabetes and no known comorbidities until one week before this admission. Prior health records were, however, unavailable to us.

**Figure 1:**
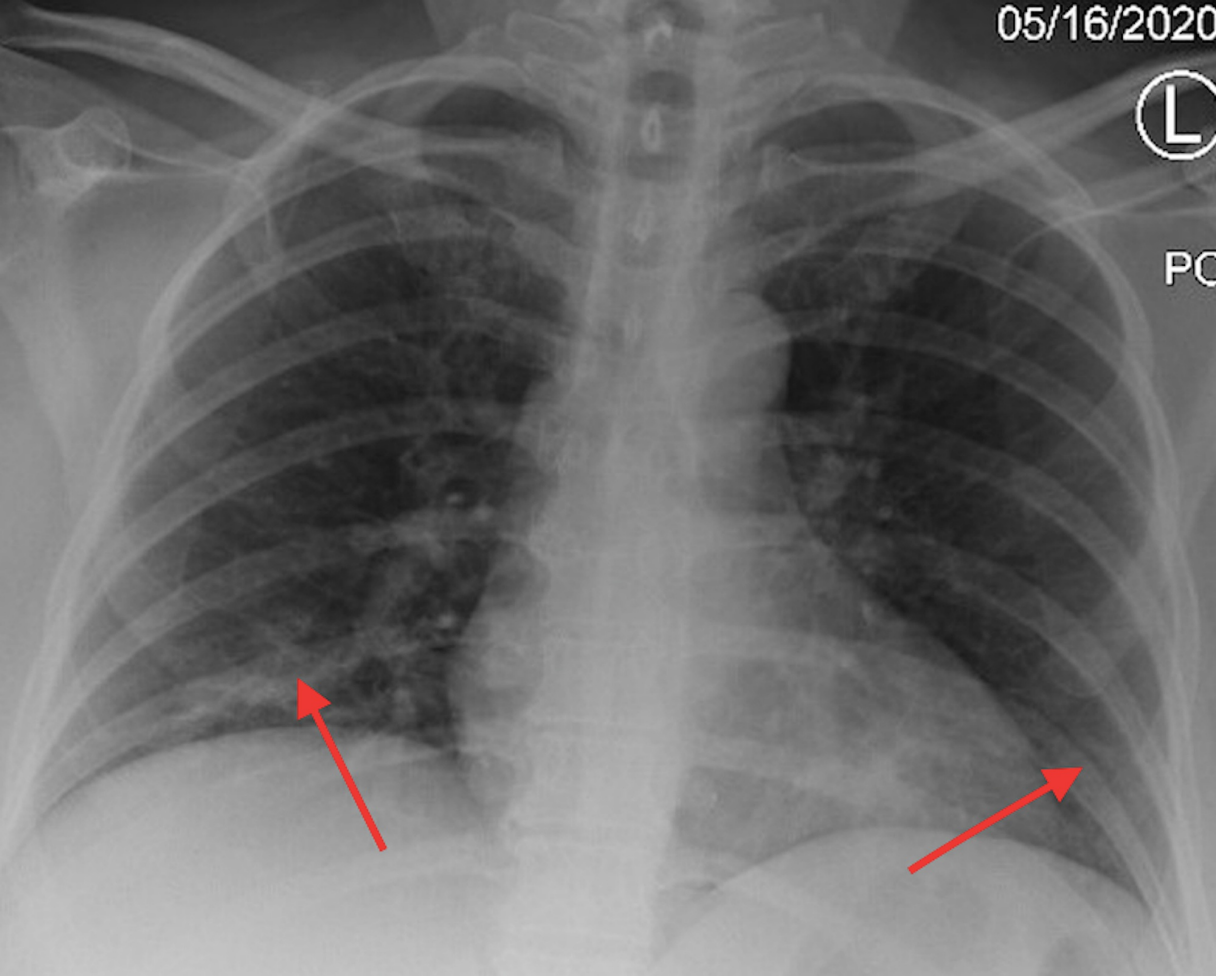
Chest x-ray on the day of admission showed mild bilateral patchy airspace opacities, nonspecific but compatible with a reported history of coronavirus disease 2019 (COVID-19) infection (red arrows)

On presentation, she was hemodynamically stable with saturation percentage of oxygen in the blood (SpO_2_) at 100% on room air. Her physical examination was unremarkable with a body mass index (BMI) of 25.39 kg/m^2^. Laboratory data were significant for high anion gap metabolic acidosis (anion gap 18, pH 7.22, bicarb 13), a blood glucose of 344, and glycated hemoglobin (A1C) of 13.7. The SARS-CoV-2 nucleic acid amplification test was used to detect COVID-19 in our patient. Inflammatory markers, including C-reactive protein, ferritin, lactose dehydrogenase, and D-dimer, were all negative. The urinalysis revealed > 1,000 proteins and > 80 ketones. Her chest x-ray showed mild bilateral patchy airspace opacities compatible with COVID-19 pneumonia (Figure 1).

As supported by clinical data, our patient was diagnosed with diabetic ketoacidosis and COVID-19. She was started on an insulin drip with the EndoTool® IV Glucose Management System (Monarach Medical Technologies, Charlotte, NC) and was admitted to the intensive care unit (ICU) for further management. She initially required high doses of insulin to normalize hyperglycemia and was later transitioned to subcutaneous insulin after spending two nights in the ICU. During this hospital stay, our patient did not require oxygen supplementation or treatment for COVID-19. There was also no evidence to support a bacterial superinfection, given the negative blood cultures and atypical bacterial workup. Due to the unusual presentation of diabetic ketoacidosis in this patient with presumed type 2 diabetes requiring high doses of insulin, further laboratory studies detecting islet antibodies and C-peptide levels were subsequently performed. As a result, elevated anti-glutamic acid decarboxylase antibody (229 IU/mL) and insulin autoantibody (46.9 U/mL) levels were noted. Along with significantly low C-peptide levels at 0.49 ng/mL, our patient was diagnosed with LADA. After educating her about her new diagnosis, she was discharged home on insulin in stable condition. 

## Discussion

COVID-19 has become a global pandemic with 57,274,018 confirmed cases and 1,368,000 deaths worldwide as of November 21, 2020 [6]. A range of symptoms was illustrated in an analysis of over 370,000 confirmed COVID-19 cases in the United States with a cough in 50%, subjective fever in 43%, myalgia in 33%, headache in 34%, dyspnea in 29%, sore throat in 20%, diarrhea in 19%, nausea/vomiting in 12%, and loss of smell or taste/abdominal pain/rhinorrhea in fewer than 10% of all cases [7]. However, as the disease spreads, we continue to discover additional manifestations and potential complications involved. A study of 161 subjects performed in Wuhan China confirmed that diabetes delayed viral clearance in COVID-19 patients [8]. Other data suggest that diabetic patients infected with COVID-19 are often associated with severe or critical illness [9]. Additionally, hyperglycemia was reported to persist after three years of disease resolution in people infected with severe acute respiratory syndrome [5]. Factors implicated in predisposing diabetics to infection with SARS-CoV-2 include increased ACE2 expression in the pancreas allowing SARS-CoV-2 entry leading to islet cell damage [4, 10], increased type-1 membrane-bound protease involved in the entry of SARS-CoV-2 into the cell, thereby facilitating viral replication [11], impaired T-cell function [12], and increased cytokine release notably interleukin 6 (IL-6) [13]. Clinical trials regarding the use of monoclonal antibodies against IL-6 (tocilizumab) to treat COVID-19 are still ongoing [5, 10, 14-15]. Lastly, it was also noted that the ability of SARS-CoV-2 to induce beta cell destruction may also predispose patients to induction of autoimmune disease and unmasking latent autoimmune diabetes in presumed type 2 diabetic patients [12]. 

LADA is a slowly progressive form of autoimmune diabetes often characterized by the presence of autoantibodies in the pancreas, diagnosis at an older age, and lack of absolute insulin requirement at the time of diagnosis [16]. Genotype analysis has shown that LADA shares numerous genetic features with both type 1 and type 2 diabetes, e.g., increased risk for HLA-DQB1 genotype with type 1 diabetes and TCF7L2 gene with type 2 diabetes [17]. The prevalence of LADA is about 10% of all diagnosed diabetes cases aged 40 to 75 years [18]. Antigen-unspecific islet cell antibodies (ICAs) and antigen-specific glutamic acid decarboxylase 65 antibody (GAD_65_) are markers of islet autoimmunity in type 1 diabetes. The presence of these antibodies in patients with suspected type 2 diabetes predicts future insulin dependency, poor response to oral hypoglycemic drugs, and an increased risk for developing ketoacidosis characterizing latent autoimmune diabetes [19]. Despite the significant prevalence of LADA, there is currently a lack of universal clinical guidelines or recommendations regarding islet autoantibodies testing. The exact duration for the development of overt dysfunction is unknown. However, one study has shown that it takes about 12 years from the time of diagnosis for patients positive with only anti-GAD_65_ antibodies and about five years from diagnosis for those with two or three positive islet antibodies to reach complete beta-cell failure [20]. Suspicion for LADA should be heightened in patients with coexisting autoimmune diseases, such as autoimmune thyroid disease, normal BMI, or poor glycemic control despite optimized therapy with oral hypoglycemics. Insulin therapy remains the standard of treatment among patients with LADA and should be initiated as soon as islet antibodies are diagnosed.

## Conclusions

LADA is considered underdiagnosed as most patients are either classified with type 1 or type 2 diabetes. There is a strong relationship between COVID-19 and diabetes as evidenced by the increased risk of hospitalization and worsened clinical course among diabetic patients infected with COVID-19. Strict glycemic control and prevention of metabolic abnormalities are important for prognosis. Therefore, the timely diagnosis of LADA sets the stage to initiate early therapeutic management.
